# Top-Down Approaches Towards Single Crystal Perovskite Solar Cells

**DOI:** 10.1038/s41598-018-23211-x

**Published:** 2018-03-20

**Authors:** Johannes Schlipf, Abdelrahman M. Askar, Florian Pantle, Benjamin D. Wiltshire, Anton Sura, Peter Schneider, Linus Huber, Karthik Shankar, Peter Müller-Buschbaum

**Affiliations:** 1 Technische Universität München, Physik-Department, Lehrstuhl für Funktionelle Materialien, James-Franck-Str. 1, 85748 Garching, Germany; 2 Department of Electrical and Computer Engineering, University of Alberta, 9211-116 St, Edmonton, AB T6G 1H9 Canada

## Abstract

Solar cells employing hybrid perovskites have proven to be a serious contender versus established thin-film photovoltaic technologies. Typically, current photovoltaic devices are built up layer by layer from a transparent substrate (bottom-up approach), while the deposition of the perovskite layer itself comes with many challenges including the control of crystal size, nucleation density and growth rate. On the other hand, single crystals have been used with great success for studying the fundamental properties of this new class of optoelectronic materials. However, optoelectronic devices fabricated from single crystals often employ different materials than in their thin film counterparts. Here, we demonstrate various top-down approaches for low-temperature processed organic-inorganic metal halide perovskite single crystal devices. Our approach uses common and well-established material combinations that are often used in polycrystalline thin film devices. The use of a polymer bezel allows easier processing of small crystals and the fabrication of solution-processed, free-standing perovskite single crystal devices. All in all these approaches can supplement other measurements of more fundamental material properties often requiring perovskite single crystals by rendering a photovoltaic characterization possible on the very same crystal with comparable material combinations as in thin film devices.

## Introduction

Organic-inorganic metal halide perovskites have undoubtedly revolutionized the field of solution-processable optoelectronics^1,2^. Apart from their favorable properties – such as high absorption and photo-emission owing to the direct band gap and good charge transport behavior – the high crystallinity of these materials is intriguing for experimentalists and theorists alike. Early after the first applications of hybrid perovskites in photovoltaic cells in 2009, first studies on macroscale single crystals were presented in 2014^3–6^. As single crystals enable studies on a more fundamental level than polycrystalline thin films, they are also useful for refinement of theoretical calculations and thus, facilitate predictions for material properties even of yet to be synthesized compounds^7–10^. To date, most studies and devices are based on organic-inorganic metal halide perovskite thin films whose wet-chemical or vapor deposition is well studied and is gradually improved in terms of reduction of defects (pin-holes, impurities), crystal size and morphology^2,11,12^. Optimized fabrication methods even allow synthesis of single crystals with minimized defect density and sizes up to centimeters while thickness can be reduced to make solution-processed wafers^6,13–16^.

Optimized devices incorporating organic-inorganic metal halide perovskite thin films with tuned band gaps are nowadays reaching power conversion efficiencies over 20% and show promising improvements in terms of long-term stability^17,18^. On the other hand, devices based on single crystals are mostly used for fundamental studies (e.g. of charge transport properties) and are seldom investigated for use as photovoltaic cells or devices in general^4,6,13–16,19–22^. Although power conversion efficiencies have generally been lower than in polycrystalline thin film devices, single crystal perovskite solar cells not only offer potentially improved long-term stability^23–25^ but also can achieve as much as 17.8% efficiency in a single crystal film grown *in situ* on a half-built solar cell stack^26^. Although a remarkable result that proves the high potential of single crystal perovskite solar cells, many investigations of fundamental properties require free-standing single crystals. To additionally test their functionality in a photovoltaic device one would have to incorporate ready-made single crystals into a solar cell stack with selective charge transport layers. To achieve this aim, we follow a top-down approach, i.e. treating ready-made perovskite single crystals in a way so that they can be used in typical solution-processed perovskite solar cell architectures. Here, we demonstrate two different approaches: (i) a p-i-n architecture with ITO\PEDOT:PSS\perovskite\PCBM(spray)\silver paste or\Al and (ii) an approach using a polymer bezel which enables a n-i-p architecture with FTO\PCBM\perovskite\spiro-OMeTAD\Au or even a substrate-free architecture with silver paste\PCBM\perovskite\spiro-OMeTAD\Au.

Perovskite single crystals, more precisely CH_3_NH_3_PbI_3_ (MAPI) and CH_3_NH_3_PbBr_3_ (MAPB), were synthesized following the inverse temperature crystallization (ITC) approach first demonstrated by Bakr and coworkers^16,22^. More details are presented in the experimental section. The as-grown crystals have a cuboidal shape in the case of MAPB and irregular polyhedral shapes in the case of MAPI owing to a preferential growth direction of the tetragonal MAPI along the $\langle 110\rangle$ crystallographic direction^27^. The advantage of this top-down approach is that the crystals can grow without physical constraints in their thermodynamically preferred shape which otherwise could introduce lattice strains^28^. On the other hand, growing a monocrystalline film *in situ* (i.e. on the substrate) during device fabrication poses certain limits to the choice of materials and was so far only successful for MAPB^29^. Finally, separating crystal growth and device built-up makes the approaches presented here more universal and easily applicable for new developments in crystal synthesis.

## Results

### Preparation of perovskite single crystals

**Figure 1:**
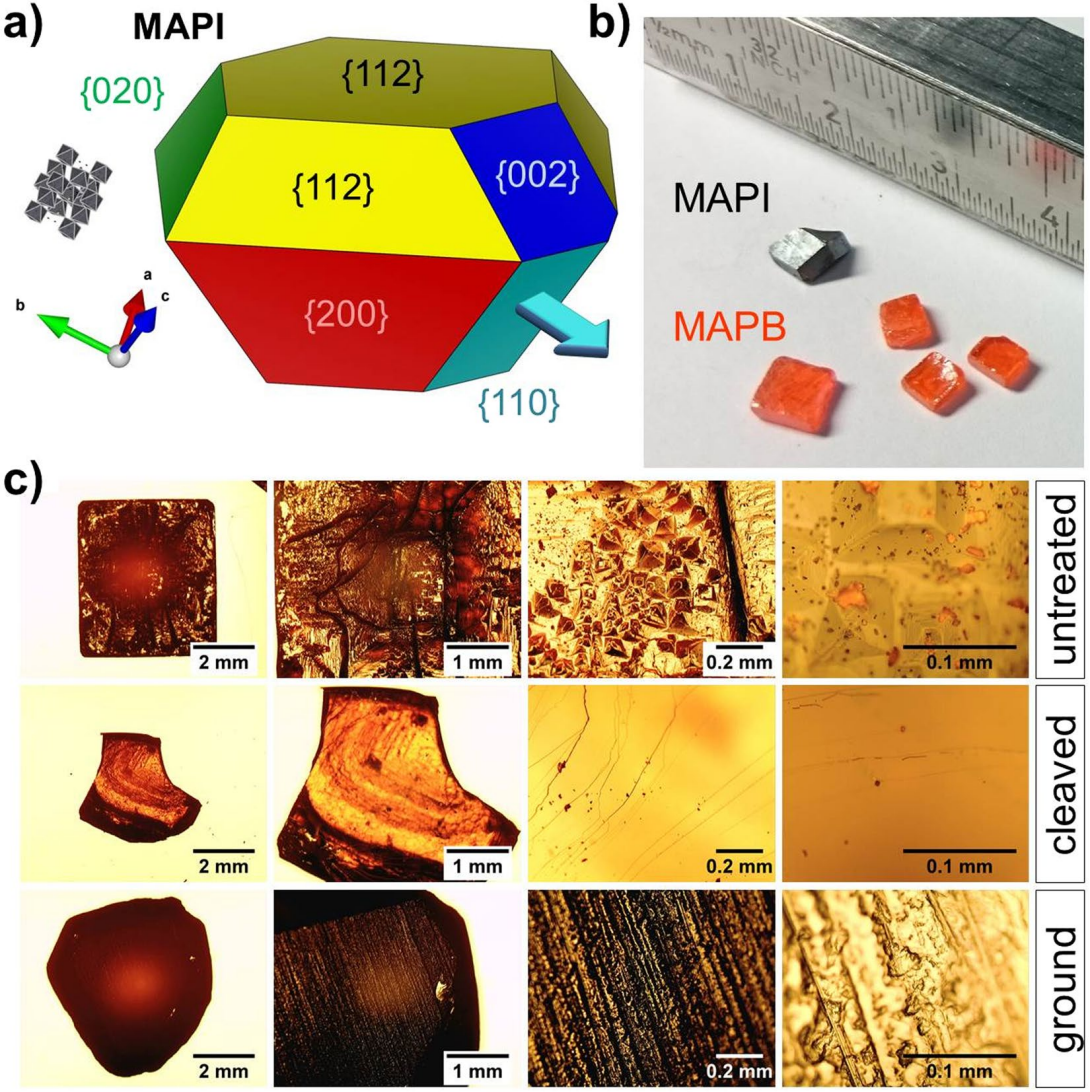
(**a**) Simulated MAPI single crystal with color-coded faces of {hkl} planes explaining the crystal shape^50,51^; a turquoise arrow points in the preferential growth direction parallel to $\langle 110\rangle$ lattice vectors^27^. (**b**) Representative samples of polyhedral MAPI and cuboidal MAPB single crystals. (**c**) Optical microscopy images of an untreated MAPB crystal (top row), a cleaved MAPB crystal (middle) and a sandpaper-ground MAPI crystal (bottom) with different magnifications.

Figure 1a correlates the faces of a typical MAPI polyhedron with the crystal planes; a turquoise arrow points in the preferential growth direction parallel to $\langle 110\rangle$ lattice vectors^27^. More possible crystal shapes are presented in Figure S1 of the Supplementary Information. In Fig. 1b representative single crystal samples for both compounds are shown. The surface of single crystals is known to be the source of defects and degradation and there are indications that structural and optoelectronic properties are remarkably different from the bulk. Thus, it is crucial that the crystal surface be treated so the crystal can be integrated into a photovoltaic device^30,31^.

Figure 1c shows optical microscopy images with different magnifications for an aged MAPB crystal (top row) where macroscale gorges, intermediate sized pyramids and tiny crystallites are observed. Short dipping of the crystals into the solvent from which they were synthesized – dimethylformamide (DMF) for MAPB and γ-butyrolactone (GBL) for MAPI – removes the outer surface, but leads to higher surface roughness (cf. Figure S2). Additionally, the subsequent fast drying of the crystal under nitrogen flow could lead to recrystallization of dissolved material on the surface. Thus, we opted for additional physical surface treatments, namely cleaving (middle row in Fig. 1c) or grinding (bottom row). Although cleaving of the crystals with a sharp scalpel leads to uniform and flat surfaces, the resulting crystal shape and size is somewhat hard to control. Especially MAPI seems to be more brittle than MAPB and often crumbles during cleaving^32,33^. Grinding with sandpaper allows precise thickness control, while additional lapping with calcined alumina lapping sheets (grit size down to 0.3 µm) reduces the crystal surface roughness.

### Single crystal solar cells with p-i-n architecture

**Figure 2:**
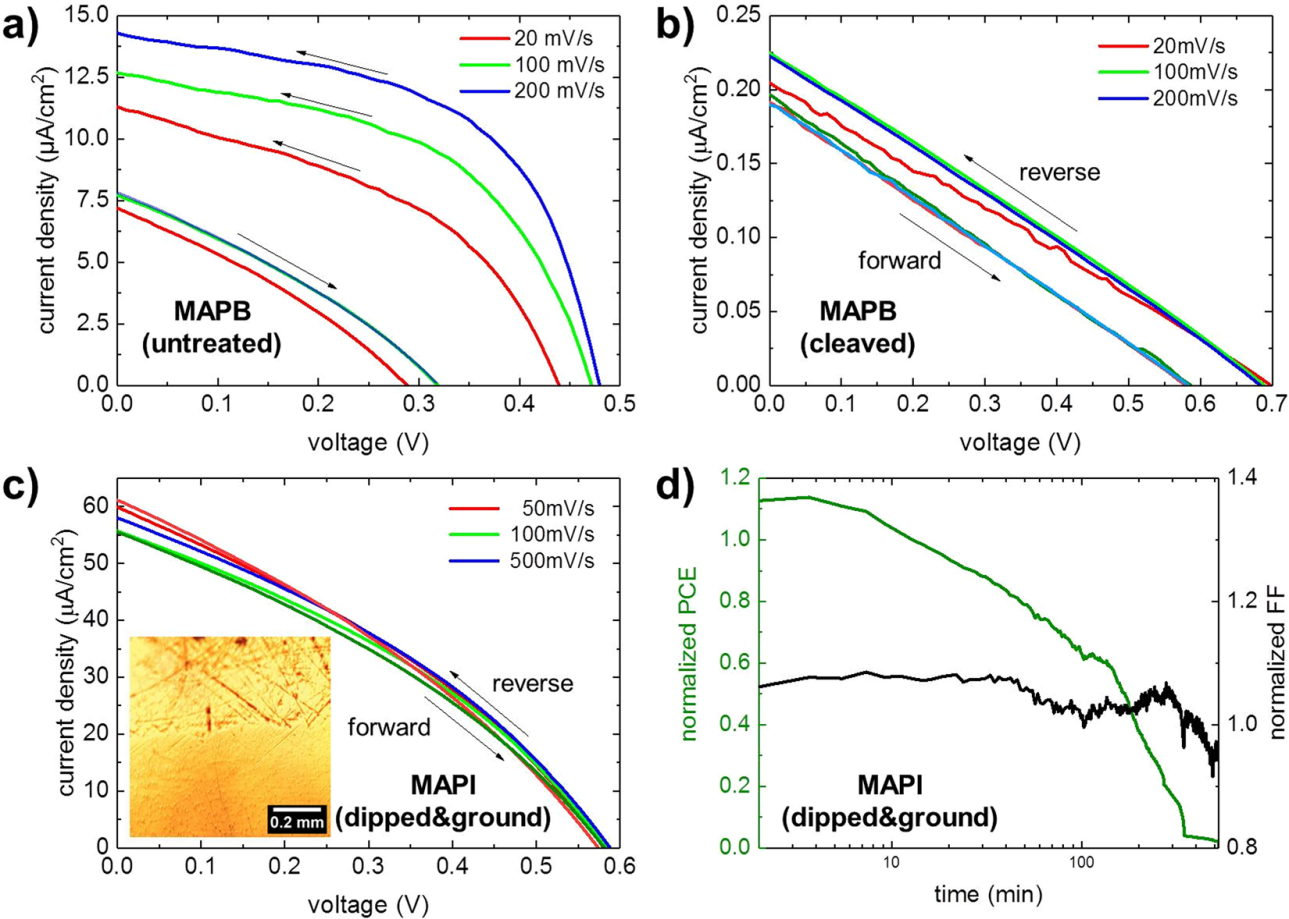
(**a**) JV curves of a MAPB (untreated) device with bias sweep in forward and reverse direction at various speeds show significant hysteresis. (**b**) JV curves of a MAPB (cleaved) device with bias sweep in forward and reverse directions at various speeds show reduced hysteresis in comparison to (**a**). (**c**) JV curves of a device fabricated from a dipped and ground MAPI crystal with bias sweep in forward and reverse direction at various speeds hardly exhibit any hysteresis. In the optical microscopy image (inset) shows a crystal surface after coarse manual grinding (upper part) as opposed to machined grinding (lower part). (**d**) Power conversion efficiency (PCE) and fill factor (FF) of the device in (**c**) over 500 min sweep at 100 mV/s every 20 s (reverse). The PCE drops to almost 0 while the FF remains roughly constant.

Single crystal perovskite solar cells with p-i-n architecture, i.e. ITO\PEDOT:PSS\perovskite\PCBM(spray)\silver paste or\Al are fabricated as follows: After cleaning an ITO-covered glass substrate via subsequent sonication in various organic solvents followed by oxygen plasma, several layers of PEDOT:PSS are deposited to achieve a hole transport layer (HTL) with sufficient thickness. Glycerol (30 mg/ml) is added to the PEDOT:PSS solution as a plasticizer so the resulting (G)PEDOT:PSS film stays sufficiently soft and the perovskite crystal can be pressed into it^34^. This formulation is used in all cases, as to not accidentally induce a work function mismatch in the HTL because the glycerol additive is known to alter the work function of PEDOT:PSS^35^. The device is completed with a spray-coated PCBM electron transport layer (ETL) and a cathode which is either conductive silver paste or thermally evaporated aluminum (cf. Figure S3). In the following, we shortly summarize the gradual improvements of this architecture, as it demonstrates some important design rules for building such devices: In a first approach to realize this architecture, an untreated MAPB crystal is pressed into a relatively thick, drop-casted (G)PEDOT:PSS film that is deposited on top of a thinner, spin-cast and annealed (G)PEDOT:PSS film. However, the resulting device exhibits strong current-voltage hysteresis and the power conversion efficiency dropped to only 60% of the initial value within the first 30 min of operation (see Figs 2a and S4). Possible reasons for the behavior are the reduced transmission though the thick PEDOT:PSS layer, short-circuits of the perovskite that pierces through the HTL as well as high charge recombination at the pristine surface of the perovskite crystal (cf. Fig. 1c). In a second approach, instead of drop-casting, two additional layers of (G)PEDOT:PSS are spin-coated onto the annealed (G)PEDOT:PSS film. Subsequently, a cleaved MAPB crystal is pressed into it, followed by spray-deposition of the ETL and thermal evaporation of the Al electrode through a shadow mask. The resulting device exhibits a reduced hysteresis in comparison to the previous approach, although the fill factor (FF) is reduced most likely due to a poor contact between the interfaces (see Fig. 2b).

In a third approach, the MAPI single crystals are dipped in GBL and then successively ground with sandpaper and lapping sheets down to 0.3 µm (see inset in Figs 2c and S2 of the Supplementary Information). The resulting solar cells hardly show any current-voltage hysteresis, independent of the sweep direction and speed. Long-term stability testing shows an improvement over the previous samples as well, while the device breakdown can be attributed to degradation or delamination of the transport layers rather than degradation of the perovskite crystal as the fill factor (FF) remains almost constant (cf. Fig. 2d)^36^. The best device fabricated with this approach yields a PCE of only 0.01% which is of course far behind the standard of polycrystalline perovskite thin films, however, measured current densities are consistent with literature for other single crystal devices^14^. Although the current densities are low they are likely to be improved with better processing of interfaces and optimization of single crystal thickness by advanced growth or lapping techniques^31,37^. Still, in the results presented here, the low current densities are the limiting factor. For example, a recent study shows a 25% loss in current density when the perovskite crystal thickness is doubled from 20 µm to 40 µm^26^. The low open-circuit voltage, however, could be connected to intrinsic properties of the perovskite that are compensated in polycrystalline thin films where various crystal orientations are present as they are consistent for this setup in our experiments (cf. Figure S5)^38,39^.

### Single crystal solar cells with polymer bezel

**Figure 3:**
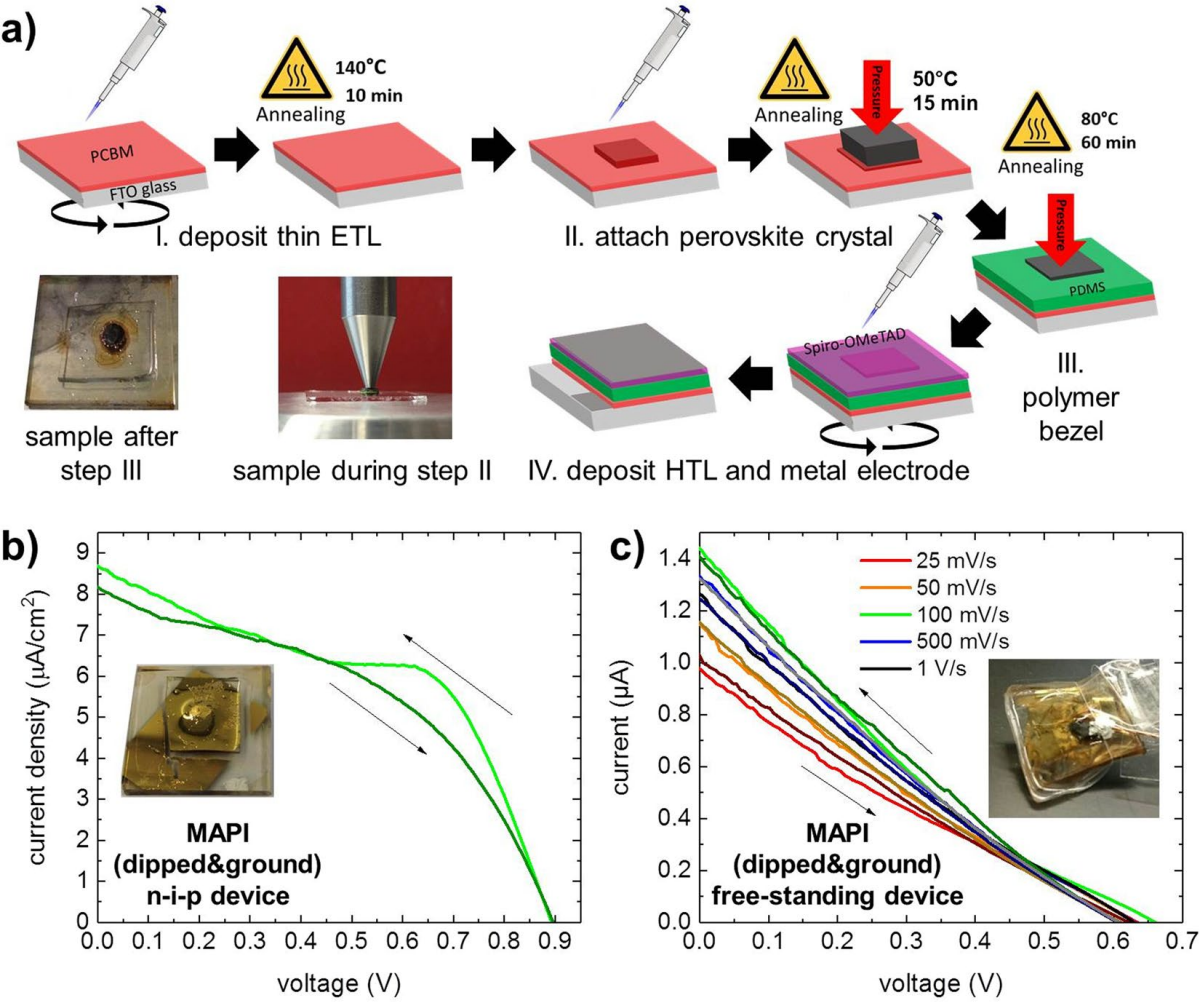
(**a**) Fabrication of a n-i-p single crystal perovskite solar cell with a polymer bezel made from PDMS. A droplet of PCBM solution is used to ensure good contact of the perovskite crystal to the underlying spin-cast PCBM layer. The crystal is then held in place during PCBM annealing and subsequent deposition of the PDMS precursor (see photographs). Finally, a spiro-OMeTAD solution is spin-cast and gold is thermally evaporated. (**b**) JV curves of a n-i-p photovoltaic device with the depicted fabrication process show negligible hysteresis at 100 mV/s scan speed and high V_OC_ as typically found in thin film devices. The inset shows a photograph of the sample. (**c**) Performance of the free-standing device still incorporated in the polymer bezel and illuminated next to the silver paste. The inset shows a photograph of the device.

Although reproducible photovoltaic devices can be fabricated with the approach presented in the previous section, the device architecture is somewhat limited by the need of a plasticized bottom layer that has the double function of mechanically fixing the crystal and taking part in the charge carrier transport process. In our case, besides restricting us to the p-i-n architecture, this approach requires rather thick PEDOT:PSS layers which are not necessary or even detrimental for efficient charge extraction. Additionally, the glycerin used as a plasticizer could attack the perovskite crystal’s surface during the imprint. In another approach, we separate these two functions by fixing the crystal with a polymer bezel produced from polydimethylsiloxane (PDMS), a cheap and commercially available material. This also makes the fabrication of n-i-p architecture possible as shown in Fig. 3a.

After spin-coating a PCBM solution onto a glass substrate coated with fluorine-doped tin oxide (FTO), a droplet of the same solution is placed in the middle while the sample is on a hot plate at 50 °C. Close to the end of the drying process, the prepared crystal is dropped into the still liquid spot. The timing of this step is crucial as the wetting of the PCBM on the perovskite crystal is quite good: If too much liquid is around, the solution could also climb the sides of the crystal. If the waiting time is too long, the PCBM layer will become too thick. The crystal is held in place by a pen-like metal bolt to avoid movement (cf. Figure S6). After an additional annealing time, an aluminum frame is placed onto the sample and the PDMS precursor solution is poured around the crystal. A solution of spiro-OMeTAD is spin-cast onto the crystal after the PDMS is fully cross-linked, and a gold electrode is thermally evaporated to conclude the device. Although the current density is lower than for the devices in the previous section, the V_OC_ is increased to 0.9 V, a value closer to what is typically observed in polycrystalline thin film devices. The hysteresis is negligible, however, the device showed strong degradation shortly thereafter and no detailed measurements could be done.

In order to fully exploit the possibilities of using a polymer bezel around the crystals, we place a perovskite crystal onto a silicon wafer and pour the PDMS precursor around it similar as described before. After the hardening of the PDMS it can be removed from the silicon wafer and the crystal surfaces are flush with the surfaces of the PDMS slab. Now, PCBM and spiro-OMeTAD solutions are spin-cast on each side and silver paste and evaporated gold are employed as electrodes, respectively. JV curves of a free-standing device embedded in the polymer bezel are shown in Fig. 3c. Although the curves show a dependence on scan speed, the scan direction does not seem to have an influence and almost no hysteresis is visible. It should be noted that the device is illuminated from the side where a small spot of the crystal is covered by silver paste. As it is unclear from which distance and depth photogenerated charge carriers can reach the silver paste electrode, the data is plotted in absolute values. The estimated current density for an active area in the order of 0.1 cm² would be comparable to the n-i-p device, although the V_OC_ is shifted to lower values again (~0.6 V). The free-standing approach has the advantage that one can remove the crystal from the bezel and illuminate it from the side (cf. Figure S7). Our device, however, shows more hysteresis and dependence on the scan speed. As the illuminated side of the crystal is not in contact with any transport layer, we attribute this to surface defects and resulting recombinations at this interface^40^.

## Discussion

We demonstrated different top-down approaches to produce low-temperature processed single crystal perovskite solar cells. In contrast to other techniques that aim to produce large-grained perovskite films by meniscus printing^41^ or roll-coating^37^, top-down approaches have the advantage that the crystal growth process is decoupled from device production. As growth processes of perovskite single crystals are optimized gradually, larger single crystal devices seem feasible^14^. Although the top-down approach is similar to established wafer-based technologies, it is not easily scalable as demonstrated recently for thin films^42,43^. However, there is a growing mass-market for small optoelectronic devices, such as in mobile electronic applications, like low-cost, self-sufficient small sensor or camera systems, or in next-generation light-emitting devices^44–46^. As it is difficult to manually grind perovskite crystals to appropriate thinness, the current densities of these devices are unlikely to be improved without proper processing techniques to achieve much thinner crystals^26,29^.

Our study shows that integration of perovskite single crystals into small, low-temperature processed devices is possible, however, proper preparation of the crystal surface is crucial^30,31^. First and foremost, these approaches can serve scientists investigating perovskite single crystals: Measurements of fundamental material properties like charge carrier mobility and trap state density^6,15,21,47^, thermal and mechanical stability^13,32,33^ or advanced structural investigations^10^ are best performed on single crystals as film morphology and grain boundaries could dominate the measurements^48,49^. Many of these studies in literature include photovoltaic measurements, although mostly with different contacts or charge transport layers as are typically used. Especially solution-processed organic contact layers are hard to deposit on the rather small perovskite single crystals. Here, the approach using a polymer bezel enables film deposition in a common way like spin-coating. Solutions with solvents that might be harmful to the perovskite crystal can be deposited on a substrate first (like the aqueous PEDOT:PSS) or deposited via spray-coating (air brushing). Thus, the here demonstrated top-down approaches will enable supplementing fundamental investigations on an existing perovskite single crystal with photovoltaic measurements in a versatile device architecture built around the very same crystal.

## Methods

### Single crystal growth by inverse temperature method

Perovskite single crystals, more precisely CH_3_NH_3_PbI_3_ (MAPI) and CH_3_NH_3_PbBr_3_ (MAPB), were synthesized following the inverse temperature crystallization (ITC) approach first demonstrated by Bakr and coworkers^16,22^. MAPI single crystals were prepared using a 1–1.3 M solution of MAPI (CH_3_NH_3_I (Dyesol-limited) + PbI_2_ (Acros-Organics)) in GBL (Sigma-Aldrich), which fully dissolved at 60 °C after 30 min of stirring. The solution is then filtered using a 0.22 µm PVDF filter. The stock solution is distributed into vials with 4 ml of solution each. The vials are kept undisturbed in an oil bath around 110 °C. After 3–4 h, the crystals were 4–6 mm in size. Upon achieving the desired size, the crystals were removed from the synthesis solution, rinsed quickly with GBL, dried under N_2_ flow, and stored in the dark in a desiccator. Similarly, MAPB single crystals were prepared using a 1.0 M solution of CH_3_NH_3_PbBr_3_ (CH_3_NH_3_Br (Dyesol-limited) + PbBr_2_ (Sigma-Aldrich)) in DMF (Fischer Scientific) which fully dissolved at room temperature. A 0.22 µm PVDF filter was used to filter the solution, then the solution was distributed into vials with 4 ml of solution each, put in an oil bath undisturbed at 80 °C. After 3–4 h of synthesis, the crystals were around 5–7 mm in size. The crystals were removed and rinsed with DMF, dried with N_2_ and stored in a desiccator in the dark.

### Preparation of perovskite single crystals

If not noted otherwise in the text, perovskite crystals were treated in the following way: First, they were dipped into either DMF (MAPB) or GBL (MAPI) at room temperature and blown dry with nitrogen. Then, common sandpaper with a grit of several tens of microns (grit designation 1000) was used to bring the crystals into shape and progress was regularly checked with an optical microscope. Further grinding was successively performed with calcined alumina lapping sheets with 1 µm and 0.3 µm grits (Thorlabs LF1P and LF03P) and care was taken so that no spot was used twice in order to prevent scratches from broken-off perovskite pieces.

### Fabrication of p-i-n architecture solar cells

Indium-tin oxide (ITO) substrates (SOLEMS Sol30) were successively sonicated in aqueous detergent solution, ethanol, acetone and isopropyl alcohol. Before further processing they were submitted to an oxygen plasma. PEDOT:PSS solution (Clevios AL4083 by Heraeus) was sonicated and filtered with a 0.45 µm PVDF filter before use. 30 mg/ml of glycerol were added by weight as a plasticizer and the solution was stirred for at least 20 min. The first layer was spin-cast at 2000 rpm for 60 s and then annealed at 140 °C for 10 min. Two additional layers were added with 500 rpm for 60 s and a second step with 2000 rpm for 10 s without annealing. The prepared perovskite crystal was then placed onto the substrate and pressed into it with copper weights wrapped in aluminum foil (605 g). The sample was then heated to 80 °C for 3 h to remove the plasticizer. A 4 mg/ml solution of (6,6)-phenyl C61-butyric acid methyl ester (PCBM) (Nano-C) in anhydrous chlorobenzene was sprayed on top at a distance of 220 mm and a flow rate of 1 ml/min with 10 sprays of 30 s each. Finally, the cathode is made up of conductive silver paste (Ferro GmbH) or thermally evaporated aluminum (~2·10^−5^ mbar).

### Fabrication of n-i-p architecture solar cells

Fluorine-doped tin oxide (FTO) substrates (SOLEMS Tec7) were successively sonicated in aqueous detergent solution, ethanol, acetone and isopropyl alcohol. Before further processing they were submitted to an oxygen plasma. A 12 mg/ml solution of PCBM in anhydrous chlorobenzene was spin-cast on top with 1500 rpm for 30 s and annealed at 80 °C for 10 min. A small droplet (<50 µl) of PCBM solution is positioned on the sample and the perovskite crystal is placed into it. A pen-like metal bolt is lowered with a micrometer screw from the top until first contact is made. As the setup is equipped with a spring, the pressure is only fixing the crystal at its position. An aluminum frame is placed onto the sample and PDMS precursor (10:1 ratio of base:curing agent, Sylgard 184 by Dow Corning) with 40 wt% n-hexane added to decrease viscosity is poured into it until the surface is flush with the crystal top surface. After curing the PDMS for 60 min at 80 °C, a 100 mg/ml solution of spiro-OMeTAD (2,2′,7,7′-Tetrakis[N,Ndi(4-methoxyphenyl)amino]-9,9′-spirobifluorene) (Solarpur® SHT-263 by Merck Performance Materials) in anhydrous chlorobenzene, with 10 µl/ml 4-tert-butylpyridine (TBP) and 30 µl/ml of a 170 mg/ml solution of bis(trifluoromethane)sulfonimide lithium salt (Li-TFSI) in acetonitrile added as dopants, is spin-cast dynamically at 800 rpm for 40 s. The samples are stored in dry environment overnight for self-doping of the spiro-OMeTAD. Finally, the anode is made up of thermally evaporated gold (~2·10^−5^ mbar).

### Fabrication of the free-standing architecture

Silicon wafers (Si-Mat) are oxidized at 1000 °C for 24 h. Prepared perovskite single crystals are placed on top and fixed with the pen-like metal bolt and as before, the PDMS solution is poured around. After the PDMS is cured, it is detached from the substrate and the PDMS slab with the crystal is sandwiched in between two sheets of silicone rubber with a hole in the middle where the crystal is sitting. The sandwich is fixed on a glass substrate for spin-coating the PCBM solution and then turned around to spin-coat the spiro-OMeTAD solution. Silver paste and thermally evaporated gold are used as contacts.

### Solar cell measurements

Solar cells performance was evaluated with a Keithley 2400 source meter and a KHS SolarConstant 1200 solar simulator (Steuernagel Lichttechnik) calibrated with a KG5-filtered silicon reference cell (Fraunhofer ISE). Due to the irregular shape of the crystals no illumination mask was employed. Instead, the sample was taken to an optical microscope afterwards and the contact as seen through the lower glass substrate was measured with the software ImageJ and used to normalize the data. This method rather serves to compensate for the different crystal sizes and to achieve comparability amongst our samples than for a standardized performance report.

### Data availability statement

The datasets generated during and/or analyzed during the current study are available from the corresponding author on reasonable request.

## Electronic supplementary material

Supplementary Information
