# Supplementary material for: Top-Down Approaches Towards Single Crystal Perovskite Solar Cells
Source: Sci Rep. 2018 Mar 20;8:4906. doi: 10.1038/s41598-018-23211-x (PMC5861077; doi:10.1038/s41598-018-23211-x)
Supplement: Supplementary file 1 — Supplementary Information [file 41598_2018_23211_MOESM1_ESM.docx]

Supplementary Information to:

**Top-Down Approaches Towards Single Crystal Perovskite Solar Cells**

Johannes Schlipf^1^, Abdelrahman M. Askar^2^, Florian Pantle^1^, Benjamin D. Wiltshire^2^, Anton Sura^2^, Peter Schneider^1^, Linus Huber^1^, Karthik Shankar^2^ & Peter Müller-Buschbaum^1^*

^1^Technische Universität München, Physik-Department, Lehrstuhl für Funktionelle Materialien, James-Franck-Str. 1, 85748 Garching, Germany

E-mail: muellerb@ph.tum.de

^2^Department of Electrical and Computer Engineering, University of Alberta, 9211-116 St, Edmonton, AB T6G 1H9, Canada

Keywords: ((maximum five, not capitalized, plural, separated by commas, no full stop))


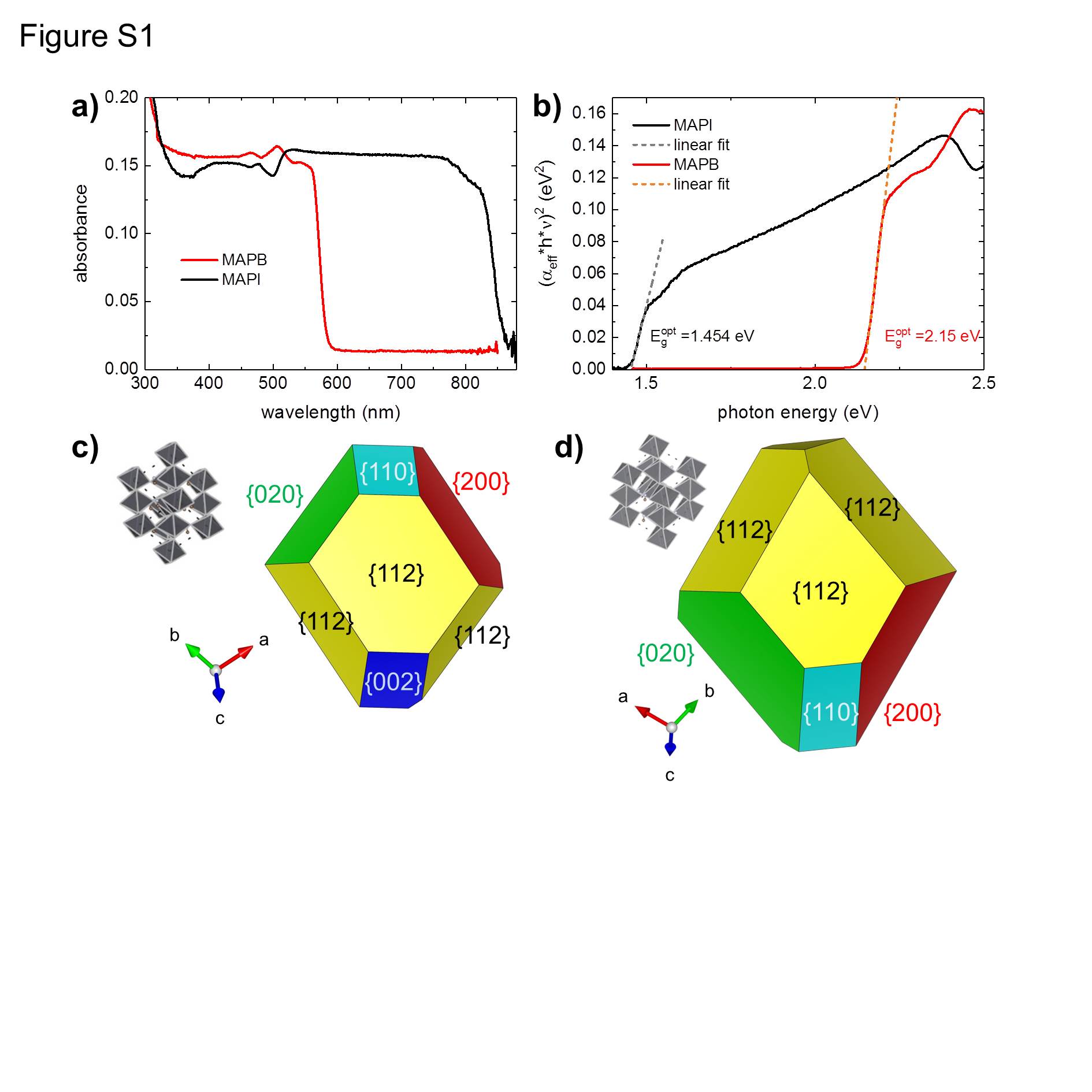


**Figure S1.** (a) Absorbance spectra of MAPB and MAPI single crystals attached into an integrating sphere (PerkinElmer Lambda 650S) and (b) respective Tauc plots showing the extracted band gaps which are slightly smaller than the values obtained for powder samples by Baikie et al.^10,45^ (c-d) Additional simulated MAPI single crystals with color-coded faces of {hkl} planes explaining the crystal shape.^32,33^


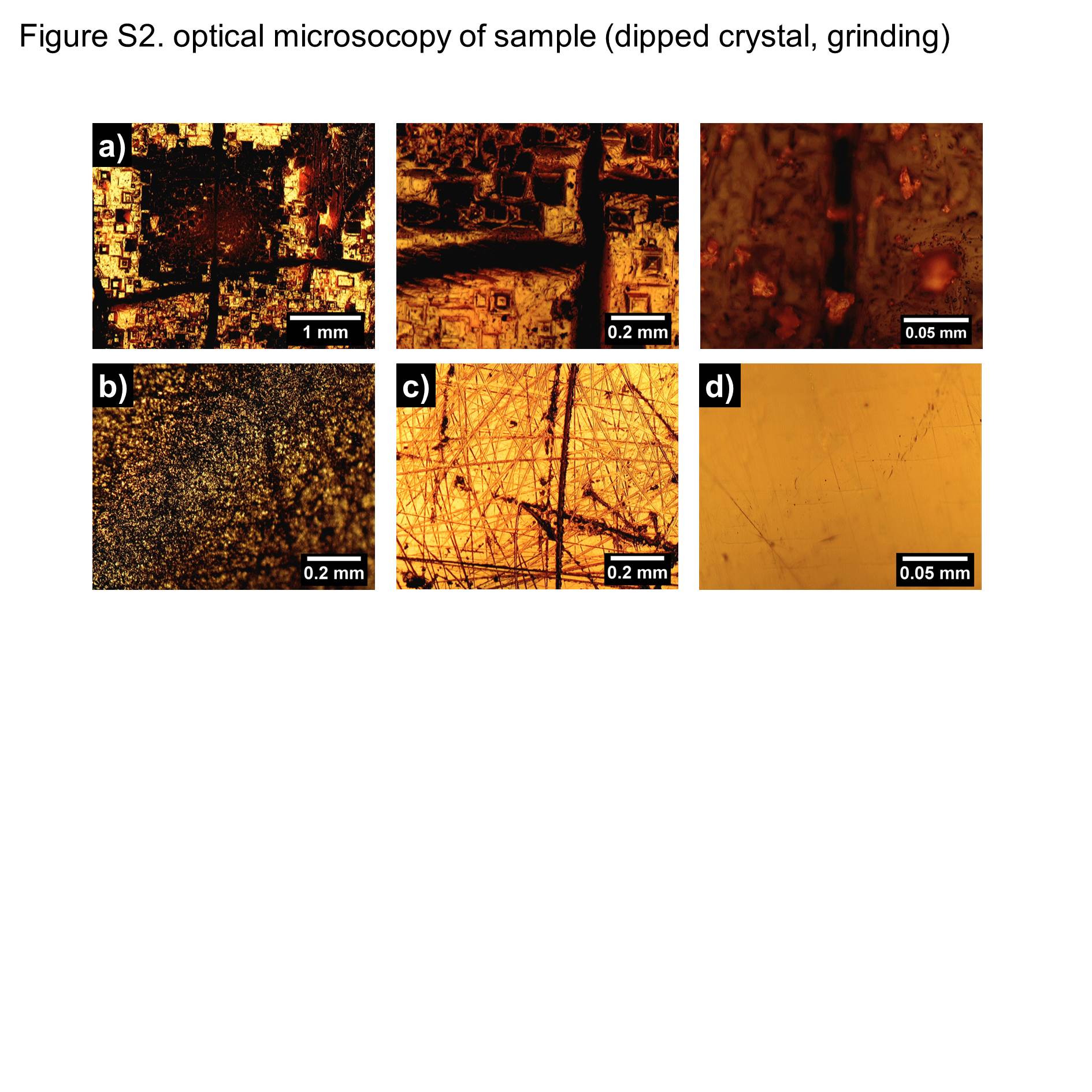


**Figure S2.** Optical microscopy images in different magnifications of (a) an aged MAPB crystal surface showing deep craters and pyramidal excrescence, (b) the roughened surface of a MAPI crystal dipped in GBL, (c) the scratched surface of a MAPB crystal manually ground with sandpaper and lapping sheets and (d) when a grinding machine is used.


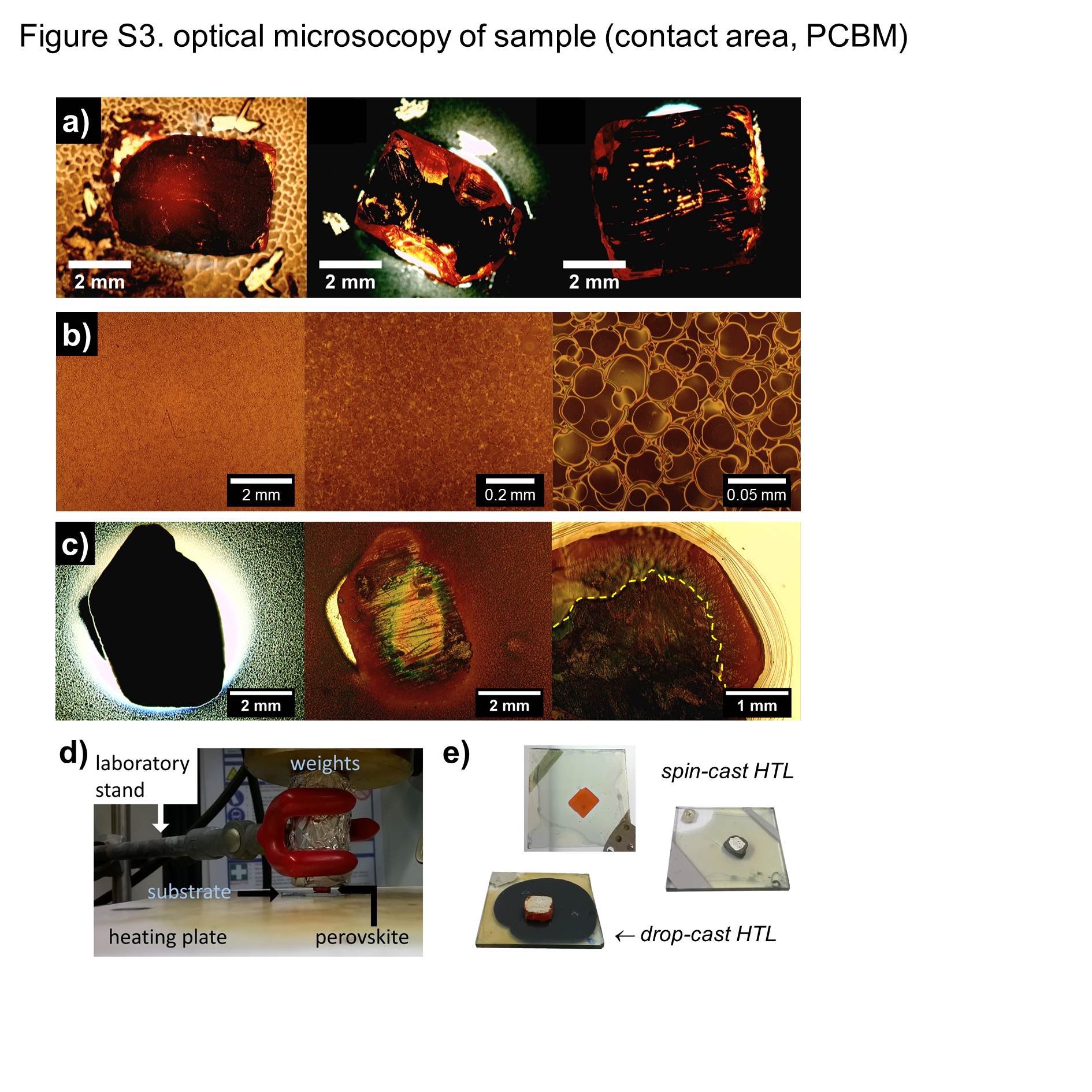


**Figure S3.** Optical microscopy images of (a) different MAPB single crystal devices – in the leftmost image, the surface structure of the spray-deposited PCBM layer is visible, (b) various magnifications of the sprayed PCBM surface, (c) various magnifications of a MAPI device from the top (left) and through the glass substrate on the bottom. The yellow line in the rightmost image marks the area of best contact between crystal and PEDOT:PSS layer. (d) Photograph of the imprint setup to attach the perovskite crystals to the (G)PEDOT:PSS. (e) MAPB and MAPI single crystal devices with p-i-n architecture on drop-cast and spin-cast PEDOT:PSS.

**
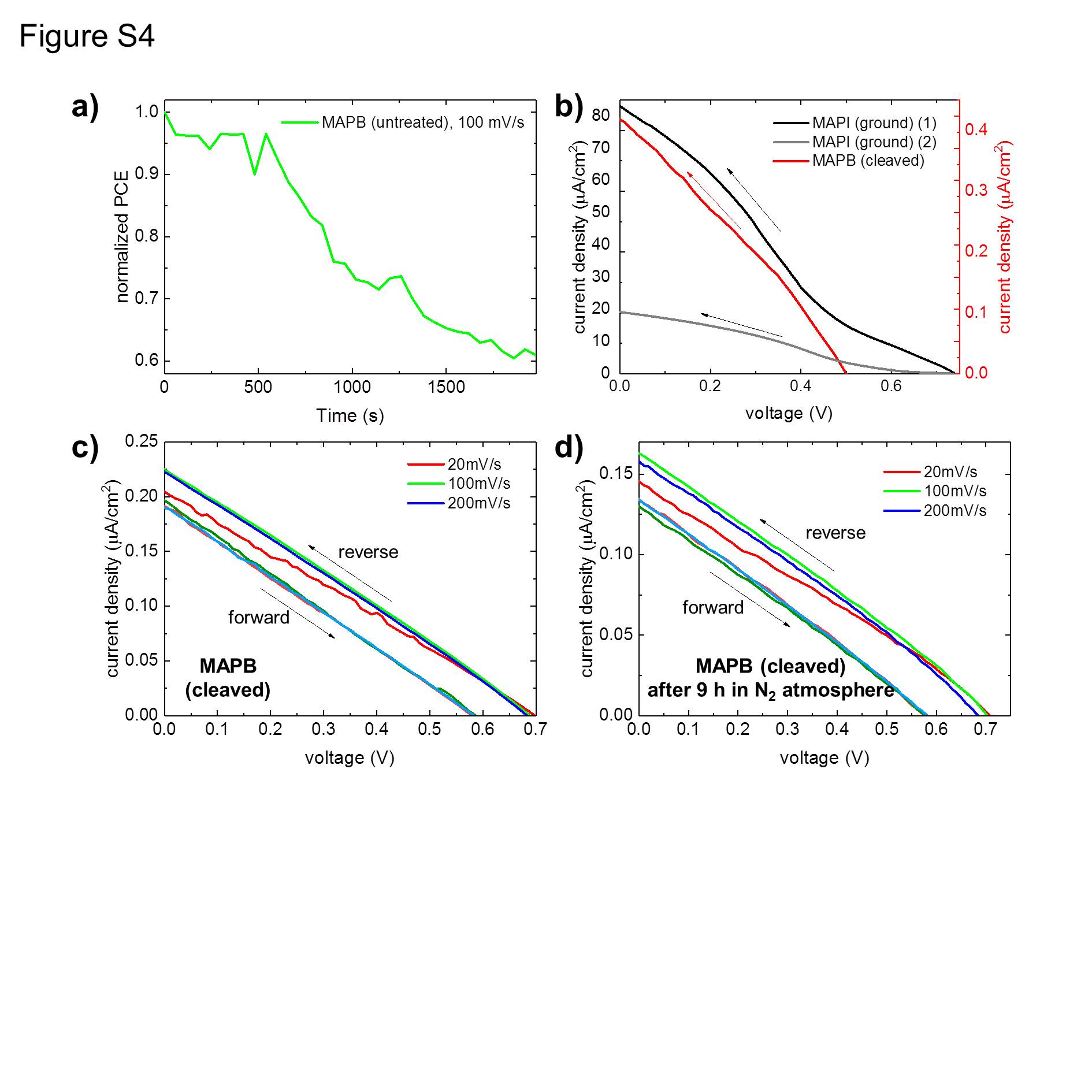
**

**Figure S4.** (a) Evolution of the normalized power conversion efficiency of a MAPB solar cell using an untreated crystal. (b) Effect of different surface treatments on the device performance of MAPB (red, right y-axis) and MAPI devices. Cleaving the crystals seems to suffer from low current, whereas grinding (1) (black, left y-axis) or dipping in DMF and subsequent grinding (2) (grey, left y-axis) leads to an S-shape. It should be noted that DMF is dissolving the MAPI too strongly, and that good results can be achieved with dipping in GBL and subsequent grinding as described in the main text. (c) JV curves of the device fabricated from a cleaved MAPB crystal with different scan velocities and directions show negligible hysteresis and (d) almost no changes in an aged sample.


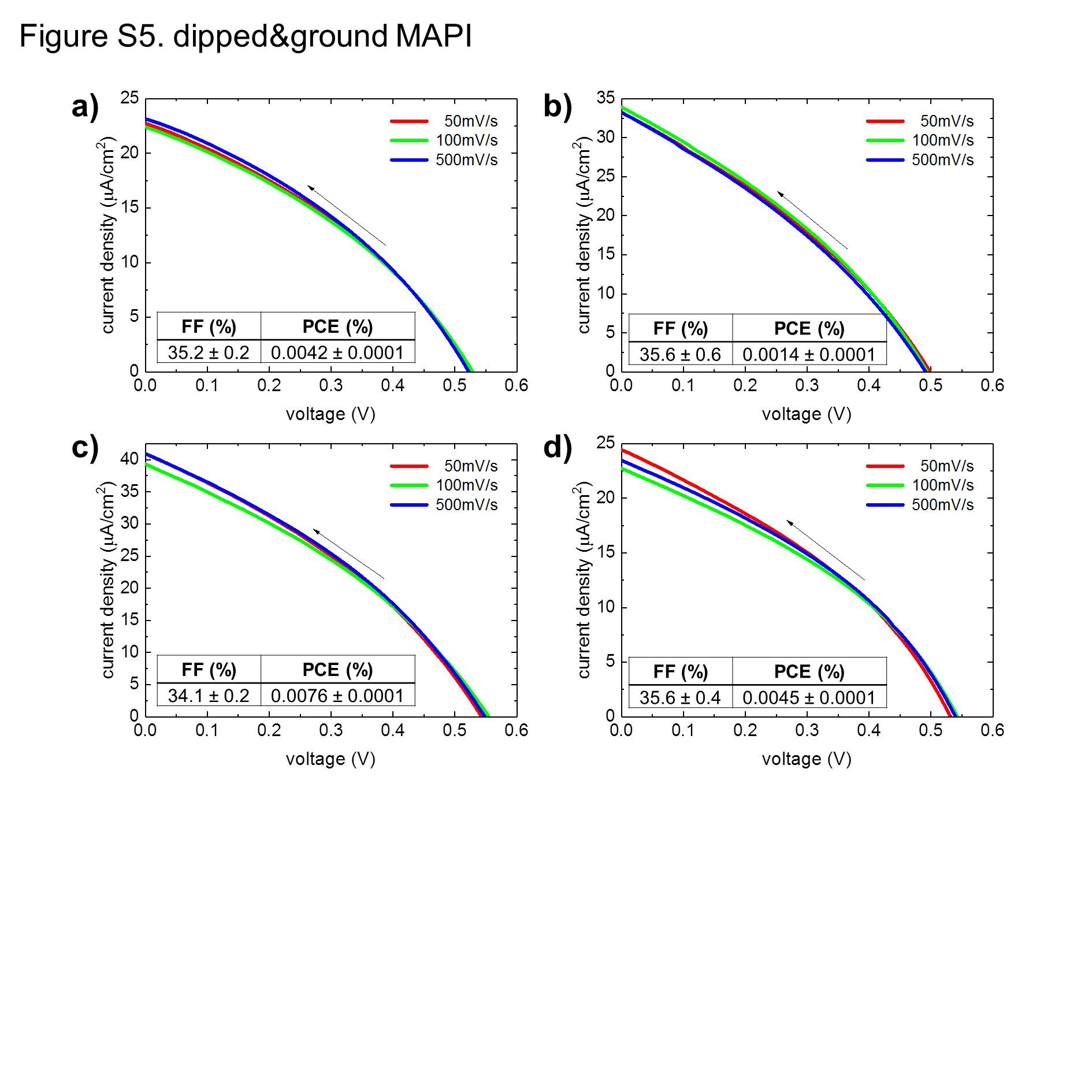


**Figure S5.** (a-d) JV curves of devices from the same batch as the device in Figure 2c, fabricated from GBL dipped and ground MAPI crystals, with bias swept in reverse direction show only marginal differences at various speeds.


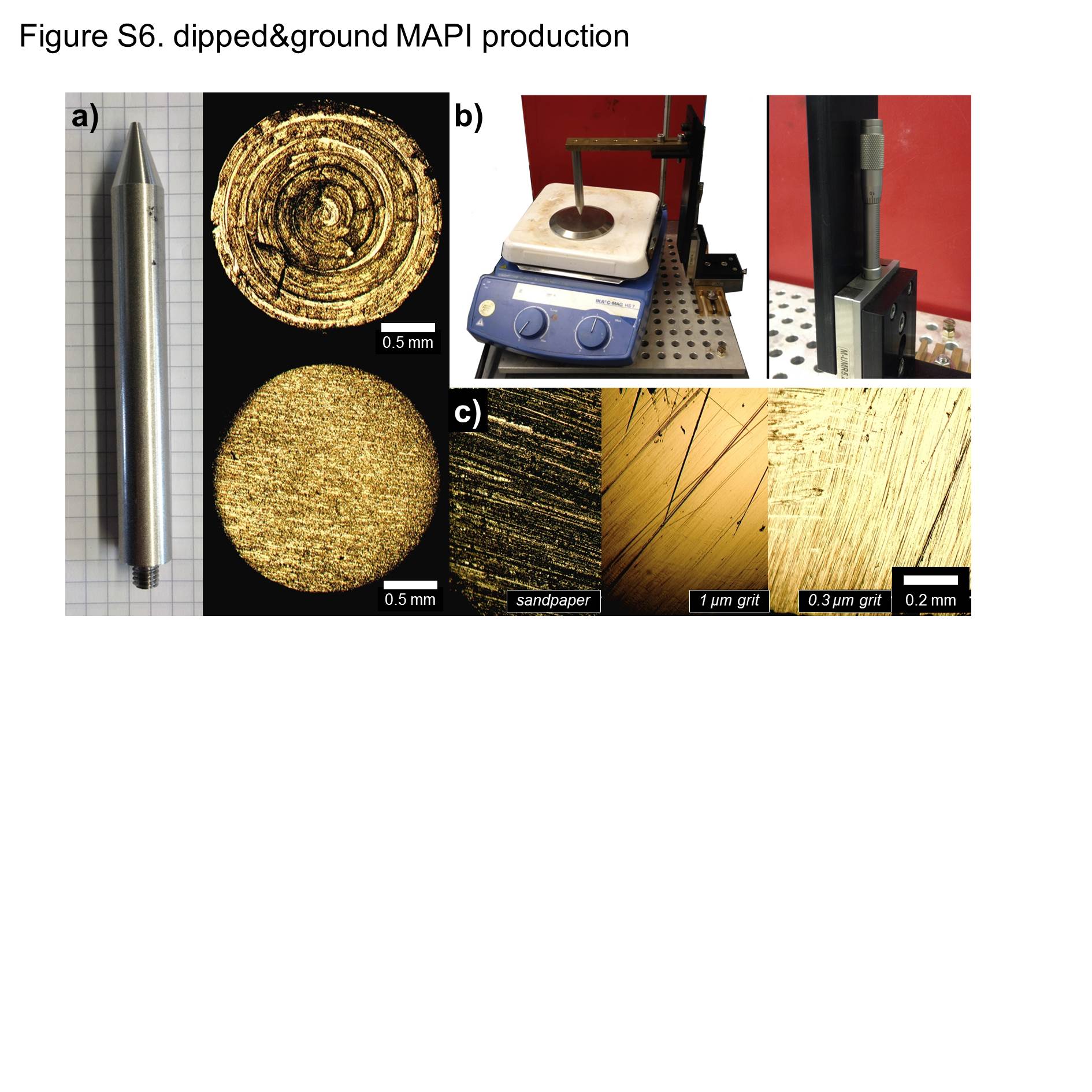


**Figure S6.** (a) Photograph of the pen-like metal bolt (left) and optical microscopy of its tip before and after grinding (right) in order to have a smooth surface and to not harm the perovskite crystals. (b) Photographs of the setup with the metal bolt installed and the spring/micrometer screw combination. (c) Optical microscopy images of the various grinding stages of a MAPI crystal as denoted after the sandpaper (grit size of various tens of microns), 1 µm and 0.3 µm calcined alumina lapping sheets.


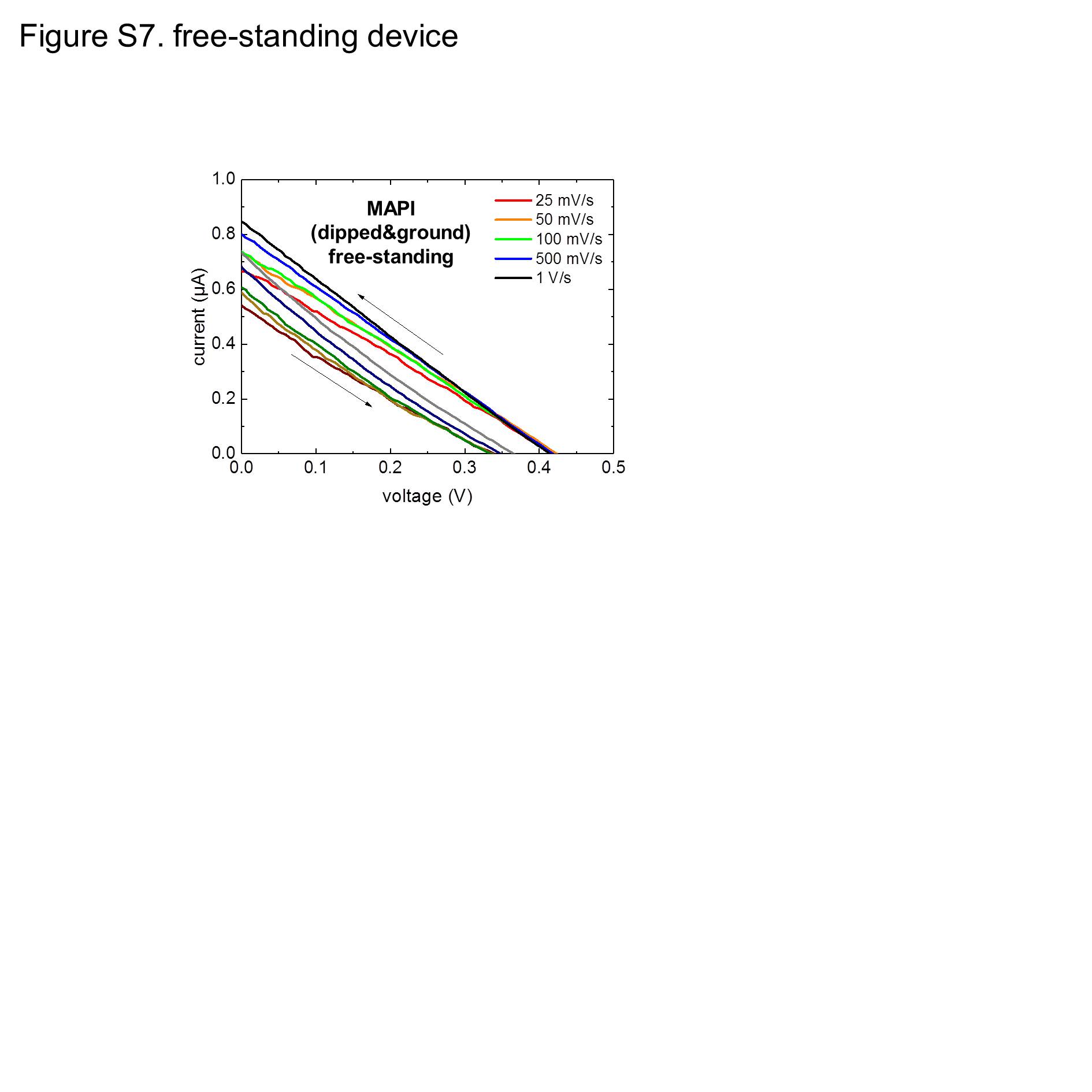


**Figure S7.** JV curves of a free-standing device with removed polymer bezel and illumination coming from the side. In contrast to the device illuminated though the ETL (cf. main text), here a strong dependence on scan speed is seen with the best performance for 1 V/s (black and grey) and the worst one for the slowest speed of 25 mV/s (red and brown). Hysteresis for fast scan speeds seems to be increased as well. As this side of the crystal is not in contact with any transport layer, we attribute this to surface defects and resulting charge recombination at this interface.
